# Observations on the Modus Operandi of Opium

**Published:** 1802-02

**Authors:** 


### *Observations on the Modus Operandi of Opium;*

[ Continued from Vol. VI. p. 478—486. ]

IT has been long known that Opium, when applied externally, is abſorbed by the lymphatics, and conveyed by the heart and arterial ſyſtem to the brain and every part of the body.

So long ago as the year 1754, Dr. Monro had found, by experiments, that a ſolution of Opium applied to the ſkin of frogs, rendered them motionleſs, and killed them in the ſame way as when applied to their interior parts.* He obſerves, (p. 306) " The effects are, however, more ſpeedy, where the doſe is equal, when the opium is applied inwardly than when applied outwardly, as might have been preſumed from the greater ſenſibility and delicacy of the inward organs."

" But the moſt important point concerning the action of opium on living animals eſtabliſhed by this author, (Dr. Monro) is its being received into the ſyſtem by means of the abſorbents; which, as far as I can judge, he proves inconteſtibly by ſhewing that the effects of opium applied to a limb, after all communication between it and the reſt of the body, by means of the nerves, is cut off, are ſoon felt in every part of the ſyſtem."

" We may indeed perceive, (ſays Dr. M.) that the effects of all the foregoing medicines (among which opium is included) when they are applied to the ſound outer ſurface of the body, are chiefly owing to their abſorption, mixture, and conveyance with the blood, ſince they operate as violently, and nearly as ſoon, when the nerves of the part to which they are applied are cut, as when they are entire."

I ſhall quote but one more obſervation from Dr. Monro's paper. It deſerves particular attention; namely, That " the effects of an over-doſe of opium, as well as of ſome other medicines, on frogs, are analagous to their effects on men and quadrupeds. †

" In other caſes when it (opium) is applied to the ſkin, whether it penetrates by inorganic pores to the viſcera of the abdomen, or if it only acts on the teguments, and particularly on the muſcles conſenting with the viſcera, we would not confidently determine; but we have certainly found the external application

* Eſſays and Obſervations Phyſical and Literary of the Philoſophical Society of Edinburgh, Vol. III. p. 297.

† An Experimental Eſſay on the Manner in which Opium acts on the living Animal Body, by Alex. P. Wilſon, M. D. p. 20, 22, 24.

application relieve the pains and ſpaſms of the ſtomach and inteſtines."*

Bergius, in his Materia Medica, aſcribes conſiderable effects to opium when applied to the ſkin. "Opium (ſays he) externe cuti applicatum, agit ſtimulando, in ſimul ſopiendo; ſi diutius ſupra cutem manet, rubedinem vel veſiculas excitat, ubi nimirum cutis tenera eſt opiumque recens."†

Dr. Crumpe expreſſes ſtrong doubts whether opium applied externally be capable of producing theſe effects, and relates an experiment which he ſeems to think deciſive. See Inquiry,‡ p. 27. But he adds, p. 29, 30, "Lind ſays, that in a caſe of Tetanus, an application of opium and camphire to the feet inſtantly removed the ſpaſm, which upon taking off the application immediately returned with its former violence; an effect which was ſeveral times produced by the repeated application of theſe medicines. Moſeley, on the contrary, who quotes this paſſage, aſſerts from his own experience, that opiates externally applied are not of the ſmalleſt utility, either in the prevention or cure of a Tetanus." ||

"Whytt, in caſes of great pain and ſickneſs of ſtomach, where the patient could not bear laudanum internally, experienced the beſt effects from its external application to the belly and region of the ſtomach. § On mentioning the matter to my friend Dr. Harding, he tells me he has ſeen two very pointed and deciſive inſtances of the efficacy of the tinct. theb. rubbed to the region of the ſtomach, in removing obſtinate vomiting, and allaying the painful and ſpaſmodic affections to which that organ is liable."

Dr. Alexander publiſhed his Theſis, entitled, "De partibus corporis animalis quæ viribus opii parent," at Edinburgh, in 1790. He attempts to ſhew, (contrary to what ſeems aſcertained by ſeveral of Dr. Monro's experiments) that opium is never received into the ſyſtem by means of the abſorbents. I think it neceſſary to mention this attempt, becauſe Dr. Alexander's experiments have been made ſince the publication of Dr. Monro's; but how unſucceſsful it is, muſt appear at firſt view to every perſon who peruſes the Theſis.¶

Could any doubt be entertained of the poſſibility of introducing opium into the ſyſtem by applying it externally, after peruſing

---

* Cullen's Materia Medica, Vol. II. p. 256.

† Berg. Mat. Med. p. 457.

‡ An Inquiry into the Nature and Properties of Opium. By S. Crumpe, M. D. p. 27.

|| On Tropical Diſeaſes, p. 494.

§ Whytt on Nervous Diſorders, c. 7, p. 362.

¶ Wilſon's Eſſay, p. 30—31.

perufing the Treatifes from which the above extracts are taken, the accounts lately publifhed in the Medical and Phyfical Journal for July, 1799, and in feveral of the fucceeding Numbers, could hardly fail, I fhould conceive, of being regarded as indubitable proofs, not only of its being poffible to introduce it in this manner, but of its producing very powerful and falutary effects, when thus applied, and in a very fhort time after its application.

Indeed, what good reafon can be given why opium may not be introduced by this mode of application as well as mercury, camphire, ardent fpirits, &c.

Without ftopping to adduce additional proofs, which I am perfuaded would now be deemed fuperfluous, I fhall go on to make a few remarks on the modus operandi of opium generally.

This is a fubject which cannot be reflected upon without folicitude. If we confult thofe authors who have favoured the public with their fentiments upon it, we fhall be ftruck with aftonifhment at the great diverfity of opinion which prevails, even amongft the ableft modern writers, who muft be allowed to be the beft authorities on the prefent queftion.

Dr. Cullen ranks opium as a fedative.* Dr. Crumpe maintains, that the medical virtues of opium are the refult of a ftimulant property alone.† Dr. Brown confiders opium as occupying the higheft place in the clafs of ftimulants.‡ Dr. Young is filent as to the modus operandi of opium, but he frequently fpeaks of its exhilarating and cordial qualities. He alfo attributes to it fuch effects as could only proceed from a fedative medicine.§ Dr. Darwin pronounces opium to be a powerful ftimulant.‖

In the firft edition of Hooper's Medical Dictionary, the words Sedatives and Stimulants are thus explained.¶

Thefe

---

* Mat. Med. Vol. II. Article Sedantia.

† Inquiry, Paffim.

‡ Elements of Medicine, Vol. I. p. 104, 1ft Edition.—"Next to ftrong drink, and immediately above it, *ftands* mufk; above it, volatile alkali; higher than this, æther; and the higheft of all, as far as experiments have yet reflected light upon the fubject, is opium."

§ A Treatife on Opium. By Geo. Young, M. D. p. 33, 47, 49, 61, and 132.

‖ Zoonomia, Vol. I. p. 80, 81, 364, &c.

¶ "Sedatives. From fedo, to eafe or affuage. Thofe medicines are fo termed, which have the power of diminifhing the animal energy without deftroying life; as *opium*, hyofciamus."

"Stimulants, from ftimulo, to ftir up. Medicines are fo termed which poffefs a power of exciting the animal energy, as wine, volatile alkali, muftard, *opium*, &c." Here opium is faid to poffefs the contrary properties of diminifhing the animal energy, and exciting the animal energy.

Thefe are not the only proofs that might be adduced; they are, however, amply fufficient to demonftrate that the queftion is ftill by no means free from obfcurity, though it has frequently engaged the attention of men remarkable for talents and medical fkill. With fuch a conviction on my mind, the moft prudent ftep I could take, would probably be to relinquifh the tafk at once; but having proceeded fo far, and feeling, as I do, the abfolute neceffity there is for its being fpeedily determined, whether opium be a fedative or a ftimulant, I fhall not be deterred from perfevering in the difcuffion, though my own infufficiency fhould prove to be the only fact clearly demonftrated.

The fubject feems to have been rendered more intricate than it really is, by the advocates for the ftimulant doctrine not adhering to a precife definition of the terms, ftimulants and fedatives.

Another inaccuracy they feem to have fallen into, is, their taking it for granted that wine and alcohol are ftimulants, and reafoning upon that fuppofition, though this is alfo a difputed point; and Dr. Cullen, after a clofe inveftigation, places them in the clafs of narcotic fedatives.* The following is an example of this mode of reafoning: "The ftimulant powers of opium are, indeed, in numerous inftances fo remarkable, that we find many writers comparing its mode of action and effects to thofe of fubftances *univerfally* reckoned powerful ftimulants, as *wine*, *alcohol*, and the whole clafs of cordials, &c."†

The

* "It is, however, as containing alcohol, that wines are to be confidered as medicines; and the confidering them as fuch we have referved for this place, in which I have put them as narcotic fedatives. That alcohol is fuch, can hardly be doubted; as, when only diluted with water fo much that it can be fwallowed, it fhows the inebriating, intoxicating, and narcotic effects of other fedatives. When taken in fmall quantity, and much diluted, it does not indeed immediately fhow its fedative power; but, on the contrary, it may appear as a ftimulant, cordial, and exhilarating liquor. As thefe operations, however, are in common to it with opium and other narcotics, they do not contradict our opinion of its proper fedative nature." Mat. Med. Vol. II. p. 315—16.——Alluding to wine, he fays, p. 317, "It is difficult, however, to fet the limits between its ftimulant and fedative powers; and if the quantity of it be gradually increafed, the latter gradually come on, and concurring with the former, produce at firft a degree of delirium or ebriety, which is generally of the cheerful kind, and which occupying the mind, excludes all thoughts of care or anxiety; but the fame fedative power carried on ftill further, renders the delirium more confiderable, and gives that irregularity and confufion of thought which is the ftate of intoxication; and at length the fedative power entirely prevailing, the animal functions, both of fenfe and motion, are gradually weakened, and the perfon falls afleep."

† Crumpe's Inquiry, p. 97, 98. See alfo Elements of Med. Vol. I. p. 192, 235, 236.

The following definitions of the terms ftimulants and narcotic fedatives, appear to me to be unexceptionable; and I muft beg to be underftood to allude to them, whenever I make ufe of either of thofe terms.

" This then is the general idea of ftimulants, that they are powers capable of increafing the mobility, and of exciting the motion of the nervous power." Cullen's Mat. Med. vol. ii. p. 131.

" Sedatives. Thefe are the medicines which diminifh the fenfibility and irritability of the fyftem, and thereby the motions and powers of motion in it." * Idem, p. 217.

I fhall firft endeavour to prove that opium is not a ftimulant.

That my remarks may be brought into as narrow a compafs as poffible, I fhall confine myfelf in the examination of this queftion, to the inquiry of Dr. Crumpe; efpecially as it contains the moft powerful arguments that have been, or I believe can be, advanced in favour of the ftimulant properties of opium.

As an apology for the liberty I am about to take, I fhall beg leave to quote the following paffage from the work of the ingenious author laft mentioned.

" That the advancement of any branch of fcience can never be retarded, either by a free inquiry into principles, however univerfally and implicitly adopted, or by the promulgation of new opinions, however ill founded, if they alfo be impartially examined, are pofitions which will appear, I imagine, upon a little confideration, fufficiently evident. For if the former be founded in truth, they will fecurely ftand the teft, and be more fully confirmed by the clofeft inveftigation; if erroneous, no greater benefit can be conferred upon the caufe of fcience, than detecting their errors and defects." Introduction, p. 1.

It will readily be acknowledged, that a treatife on the nature and properties of opium, would have been defective, had no notice been taken of its effects when applied to the tongue, eye, nofe, urethra, &c. but I by no means fee the propriety of drawing any general inference, as to the modus operandi of a medicine, *when taken internally*, from the effects it produces as a topical application.† Befides, it is highly probable, that feveral of the articles, claffed as fedatives by Dr. Cullen, and others

---

* The only writer, that I know of, who has denied the exiftence of fedatives, is Dr. Brown, Elements of Medicine, vol. i. p. 13, 14, 234, and 235. But I imagine his opinions on this fubject will hardly gain general affent, till clearer proofs are produced than have yet appeared.

† Compare the four firft experiments, Inq. p. 24—26, with p. 169.

others enumerated in Dr. Darwin's catalogue of Torpentia, would produce pain, a ſenſe of heat and of inflammation when applied to thoſe parts, in as great a degree as opium. That this is the caſe with reſpect to Camphire, appears from the following:

Exp. 1. I introduced a ſmall quantity of a watery ſolution of camphire into my right eye. It immediately occaſioned conſiderable pain, and an effuſion of tears, which continued ſix or eight minutes, and did not go off entirely in leſs than fifteen; the tunica conjunctiva appeared a good deal inflamed.

Exp. 2. I introduced a ſmall quantity of a watery ſolution of opium into my left eye. The pain was very little more ſevere than that which I experienced from the camphire; it ſubſided rather ſooner; the inflammation was not greater than in the firſt trial.

Exp. 3. I introduced a ſmall quantity of a watery ſolution of tobacco into my right eye, which had been ſubjected to the action of the camphire. It immediately occaſioned moſt acute pain, and a copious effuſion of tears, which continued eight or ten minutes.

Exp. 4. I introduced a ſmall quantity of the ſolution of tobacco into my left eye, which had been ſubjected to the action of opium. It did not occaſion nearly ſo much pain as when it was applied to the right eye, but a good deal more than either the opium or camphire had occaſioned.*

The three firſt experiments were repeated upon my houſe pupil without any difference in the reſult, except that the camphire cauſed more pain than the opium; but it is proper to mention that the eye to which the camphire was applied was a little inflamed at the time.

It appears from the firſt and ſecond of the above experiments, that very little can be inferred, in favour of the ſtimulant qualities of opium, from the firſt, ſecond, third, and fourth experiments related in the Inquiry.† The third and

* The ſolutions uſed were of the ſame ſtrength as the ſolution of opium uſed by Dr. Crumpe, namely, in the proportion of one drachm of each article to two ounces of water. It ſhould not be omitted that two drops of ſp. of wine were added to the camphire; but the trituration was continued ſo long, that the whole, or very nearly the whole, muſt have evaporated.

† The evidence produced by Dr. Cullen, in favour of the ſedative powers of camphire, appears to me to be unanſwerable. Mat. Med. vol. ii. p. 293—298.

Dr. Crumpe ſays, (Inq. p. 28,) "The authority however of a late moſt reſpectable character, Dr. Cullen, *whoſe practical remarks ſhould ever be looked up to*, would lead us to conclude, &c."

fourth prove, (what was already known) that opium diminishes the fensibility of the parts to which it is applied.

Dr. Crumpe's fifth experiment, (p. 27) cannot now be admitted as deciding the point for which it was inftituted.

The following accurate defcription is given (p. 30, 31) of the effects refulting from the internal ufe of opium.

"The effects of opium taken internally come next to be examined; and as their great variety requires fome arrangement, I fhall confider it as affecting, 1ft. the vital; 2d. the natural; and 3d. the animal functions.

From an adequate dofe of opium, the following changes are obfervable in the vital functions. The pulfations of the heart and arteries are firft rendered quicker, fuller, and ftronger, and afterwards flower than at the time of taking it. With the increafe of frequency in the pulfe, the heat of the body is generally fomewhat augmented; the refpiration is little affected, except a large dofe has been taken; towards the conclufion of the operation of which it becomes flow, ftertorous, and laborious."

Dr. C. then quotes feveral paffages from different authors, to prove that the *primary* operation of opium taken internally, is to accelerate the pulfe, and excite the force of the circulation; and afterwards relates three experiments, which he made to afcertain this point, and finds it fully confirmed.

It is admitted by all parties, that the *primary* operation of opium, taken internally, is to produce the effects abovementioned; but it is evidently proved, in my opinion, by Dr. Crumpe's experiments, that it acts *ultimately* and *principally* as a fedative.

As this is a point of great confequence, I fhall dwell a little upon it.

The 7th, 8th, 17th, and 19th experiments, are thofe to which I particularly allude at prefent.

Experiment 7.* "At one o'clock, P. M. I gave to a robuft healthy young man, whofe pulfe beat but 44 in a minute, its natural ftandard, one grain of opium diffufed in a fmall quantity of warm water. He had never before taken any of the medicine, and his pulfe was affected in the following manner." †

It continued to beat 44 till 20 minutes had elapfed.

"After 25 minutes had elapfed, there was a manifeft increafe as well in the ftrength and fulnefs, as in the frequency of the

* Inquiry, p. 34—35.

† Dr. Crumpe's fcale cannot be introduced here conveniently; but the alterations which occurred may be rendered fufficiently intelligible without it, the pulſe. In an hour this began to diminiſh, and continued decreaſing till near the end of the experiment. A ſlight heavineſs, which came on 55 minutes after he had taken the opium, was the only other effect experienced from it."

But I wiſh particularly to point out the alteration in the pulſe, marked in the ſcale as having taken place in 90 minutes after the opium had been taken. It appears that the pulſe was then only 42, and the ſame in 100 minutes; in 110 minutes it was only 40, and the ſame in 120; and did not return to its natural ſtandard, 44, till 135 minutes had elapſed.

The 8th, 17th, and 19th experiments, prove *decisively* the ſedative power of opium. The doſes taken were larger, and the *primary* and *ultimate* effects are more clearly marked.

Exp. 8, (p. 35) "Forty-five minutes after twelve, P. M. my pulſe beating 70 in a minute, I took two grains and a half of opium diſſolved in an ounce of water."*

"In 20 minutes perceived a ſlight warmth, and ſoon after a degree of moiſture on my ſkin, the fulneſs of pulſe increaſing as well as its frequency. In half an hour I found myſelf, or at leaſt imagined myſelf, more alert and ſprightly than before; *in* 40 *minutes perceived a pleasing kind of languor gradually increasing*; *in* 90 *minutes a dull head-ach*; *in two hours time the head-ach was much increased, and attended with drowsiness and nausea*; *in two hours and a half every disagreeable symptom was increased, my pulse* 70; *took a spoonful of vinegar, which somewhat relieved the nausea*; *in two hours and three quarters found all the above symptoms still increasing, and attended with slight vertigo, and tremors in my hands*; *pulse the same as before. In three hours and a half the nausea was considerably augmented, the other symptoms as before, and I at length threw up the contents of my stomach. The head-ach and vertigo were soon after relieved*; *but I continued in a stupid state for the remainder of the day.*"

Now, if the ſymptoms juſt enumerated are to be admitted as proofs that opium is a ſtimulant, I am clearly and decidedly of opinion, that it ought *never* to be given with a view to its ſtimulant effects.

Dr. Crumpe's pulſe beat 70 when he took the opium, and the ſcale ſhews that in 5 minutes after, it beat 74, and continued to do ſo till 15 minutes had elapſed. At the expiration of 20 minutes, it beat 76; in 25 minutes, it beat 78, and in 30 minutes 80, the higheſt number it arrived at; in 35 minutes 72; in 40 minutes 70; in 45 minutes it was reduced to 64;

 in

* The ſcale is omitted here.

in 50 minutes it continued 64; in 55 minutes it beat 66; in 60 minutes 70; in 75 minutes 70; and in 90 minutes 70.

"These experiments, and the authorities above quoted, seem sufficiently to evince, that the *primary* effect of opium on the pulse is to accelerate and render it fuller; and we can only account for the mistake of those who maintain a contrary opinion, by supposing that they neglected to examine the state of the pulse *shortly* after the opium had been taken, attending only to the ultimate changes it underwent;* and this supposition will receive further confirmation from considering, that the only favourer of this opinion, who, as far as I know, has given a particular detail of any experiments in its support, has totally omitted any account of the state of the pulse during the first half hour;† although, as sufficiently appears from those above related, its frequency is, during that period, more augmented than during any other, and indeed in a shorter time than has been generally imagined. After this increased frequency of pulse has continued for some time, it again becomes slower; and this change, as may be observed in the eighth experiment above stated, and as I have remarked in many others of a similar nature, is frequently *very sudden*. The number of pulsations, in a given time after this change has once taken place, continue to diminish, and at length, if the dose has been any way considerable, fall far short of the natural standard of health; the difference in this respect being generally proportioned to the quantity of medicine which has been taken, and the consequent increase of quickness occasioned by it in the heart's pulsations. If the dose has been so great as to induce death, intermissions in the pulse have been generally remarked before that event took place."

"With the increased frequency of pulse, the heat of the body is, as above-mentioned, somewhat augmented; at least, in all my experiments I found this to be the case, if I could judge by my own feelings, and it was sometimes attended with flushings of the face. I could perceive little alteration in the respiration except the dose had been considerable; by such it was in the end rendered somewhat laborious. Those who have

* Has not Dr. C. fallen into a similar mistake, and one which may produce infinitely worse consequences, by having attended so entirely to the *primary* operation of opium, as not to have allowed the *ultimate* operation to have any influence in his decision? This would have appeared less extraordinary, if he had not described the ultimate effects with so much accuracy and impartiality, as he has every where done.

† Bard. Dissertatio inauguralis de opio, Edinburgi edit. An. 1763.

have had an opportunity of remarking the ſymptoms which preceded death, from an over doſe, have obſerved it ſlow, ſtertorious, and laborious."

"The natural functions are affected by opium in the following manner: The appetite and digeſtion, from unuſually large, or frequently repeated doſes, are generally impaired, and vomiting often induced; the diſcharges from the inteſtines are diminiſhed or ſuppreſſed; ſecretion and excretion are impeded in every part of the ſyſtem, except the ſkin, the diſcharge from which is evidently augmented."

The following obſervations (p. 39,40) as far as I can judge, militate ſtrongly againſt Dr. C's theory.

"Theſe effects are generally known and acknowledged to reſult from the operation of opium. The eaſtern nations are ſo well convinced, by experience of its powers in *diminiſhing the appetite*, that, in the famine which prevailed in the Eaſt Indies in the year 1770, the wretched ſufferers purchaſed it at exorbitant prices, to allay the cravings of hunger,* and ſmooth the approach to death. That its too free uſe impairs digeſtion and induces vomiting, is well known; but all theſe effects would ſeem only to ariſe from an over-doſe of the medicine, or from its repeated and conſtant exhibition. Its powers in *leſſening the inteſtinal evacuation* need no proof. *That it diminiſhes the ſecretion of the mucus which moiſtens the mouth and fauces, is evident from the thirſt it is productive of*; and that the ſecretion, or, more probably, the excretion of other fluids are impeded by it, is at leaſt probable, the gall and urinary receptacles having been

* If ſtimulant medicines be indicated in ſuch caſes, why ſhould we not have recourſe to ſuch as do not produce diſagreeable or dangerous conſequences, when taken in moderate quantities; as horſe-radiſh, or muſtard; or the reſinous gums, as guaiacum, myrrh, &c. or to ſome of the aromatics, as cinnamon, pepper, ginger, cardamom ſeeds, ſnake-root, &c. the eſſential oils or oxygen gas. I imagine there are few perſons who are in that unhappy ſituation, that would not prefer any of the above-mentioned ſubſtances to opium, were it likely to have the ſame effect. Is not the evidence in favour of the ſtimulant properties of the articles I have enumerated much more *deciſive* than that in favour of the ſtimulant properties of opium; and do they not produce effects in almoſt every reſpect different and even oppoſite to thoſe produced by opium? *If opium impairs the appetite and digeſtion, induces vomiting, diminiſhes or ſuppreſſes the diſcharges from the inteſtines, and impedes ſecretion and excretion in every part of the ſyſtem except the ſkin*; *and if the articles enumerated above excite and improve the appetite and digeſtion, allay vomiting, increaſe the diſcharges from the inteſtines, promote ſecretion and excretion in every part of the ſyſtem*; theſe circumſtances alone are ſtrong preſumptive proofs (though I think they might with more propriety be called demonſtrative proofs) that they *cannot* belong to the ſame claſs as opium.

been found in thofe animals to whom confiderable dofes were given, replete with their refpective fluids; and the generality of writers agreeing in pointing out thefe as amongft its moft conftant effects."

"In the animal functions, the principal alterations occafioned by opium are the following: (p. 43, 44.)

The hilarity of mind is by degrees augmented, and continues to increafe, if the dofe has been confiderable, until the delirium of intoxication is produced; which, as when refulting from *fpirituous liquors*, is attended in different conftitutions with different fymptoms. It is, however, more generally productive of a pleafant and joyous ftate of mind than the contrary; and it, in many, occafions an increafed *difpofition* to venery. After thefe effects have continued for fome time, *they are fucceeded by others of a very oppofite nature*; *the mind becomes gradually dull and languid*, *the body averfe to motion*, *little affected by cuftomary impreffions*, *and inclined to fleep. If the dofe has been confiderable*, *all thefe fymptoms continue to increafe*; *and tremors*, *convulfions*, *vertigo*, *ftupor*, *infenfibility*, *and depri-vation of mufcular action appear varioufly complicated*, *and in various degrees*, *proportioned to the excefs in the dofe*, *and peculiarity of conftitution in the fufferer*."

We meet with more evidence of the fame kind, (p. 45 to 53) but which is ftill liable to the fame objections as have been already made; and great ftrefs is laid on an experiment made by Dr. Ramfay: but here alfo "*the ufual confequences of inability of motion*, *difturbed fleep*, *lofs of appetite*, *and at length convulfions followed*." (p. 48.)

"In the eaftern countries, (p. 48.) where opium is taken in very large quantities, its enlivening and exhilarating effects are univerfally known and acknowledged." But are not its *ultimate* effects every where the fame?

"The Turks, and other nations, fwallow it in large quantities, when marching to battle, or under any other circumftances which require a mind void of depreffing fears, and infpired with fortitude."

This opinion is a very prevalent one; but here I would appeal to facts, and enquire whether opium does really, in thefe inftances, produce a mind *void of depreffing fears and infpired with fortitude?*

May not fuch an effect, (fuppofing it for a moment really to take place amongft the Turks, and in the eaftern countries alluded to,) be owing in part to their Predeftinarian principles? Their great inferiority, however, when oppofed to European foldiers, cannot arife, *folely*, I fhould imagine, from want of difcipline; pline: and what can account more ſatisfactorily for that inferiority, than the habitual uſe of a drug ſo debilitating as opium?

"If oppreſſed by cares or misfortunes, they have recourſe to the ſame aſſiſtance; and from its exhilarating powers experience a temporary ſuſpenſion of melancholy and anxiety. In ſhort, like wine and ſpirituous liquors in civilized Europe, it is in theſe countries the ſupport of the coward, the ſolace of the wretched, and the daily ſource of intoxication to the debauchee."

In many of theſe caſes its exhilarating effects may probably be explained in this way. The too frequent conſequences of anxiety and misfortunes are, loſs of ſleep, want of appetite, inactivity, head-ach, &c. followed by increaſed excitement in the brain, and terminating, ſometimes in ſynochus, ſometimes in typhus. The ſoothing and ſalutary effects of opium in ſuch caſes are univerſally known and acknowledged.

The following is, I believe, a juſt deſcription of the conſequences reſulting from the habitual uſe of opium. Inq. p. 51 and 52.

The Baron de Tott is very particular in his account of the effects of opium on the Turks. Speaking of thoſe who give themſelves up to its immoderate uſe, he ſays, "Deſtined to live agreeably only when in a ſort of drunkenneſs, theſe men preſent above all a curious ſpectacle, when they are aſſembled in a part of Conſtantinople called Teriaky Icharchiſſy, the market of opium eaters. It is there that, towards evening, one ſees the lovers of opium arrive by the different ſtreets which terminate at the Solymania, whoſe *pale and melancholy countenances would inſpire only compaſſion*, did not their ſtretched necks, their heads twiſted to the right or left, their back bones crooked, one ſhoulder up to the ears, and a number of other whimſical attitudes, which are the conſequence of this diſorder, preſent the moſt ludicrous and the moſt laughable picture."

I ſhall cloſe my remarks, for the preſent, by relating the reſult of two experiments, which I have lately made.

In my laſt paper, inſerted in the Medical and Phyſical Journal for December, 1801, I expreſſed myſelf as follows.

"Hitherto, my attempts to aſcertain the *primary* operation of opium, applied externally, on the pulſe, have not been ſucceſsful. Patients afflicted with ſpaſmodic or convulſive diſeaſes being uſually timid and apprehenſive, are not favourable ſubjects for the purpoſe; but I have reaſon to believe opium acts more directly and ſimply as a *ſedative*, when applied externally, than when given internally; and I think it is principally, if not entirely, owing to this difference in the modus operandi, that the the superior advantages of applying it externally, in certain cases, are to be attributed."

Since that time I have put this interesting question to the test of experiment in the following manner:

They were made upon Mr. J. Hall, my house pupil, who is sixteen years of age, healthy, but not robust. I have frequently examined his pulse, and always found it, except when hurried by exercise, fluctuating between 90 and 108. Every circumstance likely to render the result doubtful was carefully avoided. He sat in an easy upright posture from the commencement to the conclusion of each experiment. The time chosen for the first experiment was six o'clock in the afternoon, when four hours had elapsed since he had taken any kind of nutriment; he had kept still and quiet all the afternoon, and the room was of a moderate temperature. The preparation used was the following:

℞. Tinct. opii. drachm. duas. ol. olivar. drachm. tres, vit. ovi. drach. unam. M. which was rubbed on the right leg and top of the foot till the whole was absorbed; the hand of the person who applied it being covered with a soft oiled bladder.

Immediately before the experiment commenced his pulse beat 108, firm and regular, and the following alterations took place in the different functions:

| | Min. | Pulse. | |
|---|---|---|---|
| In | 10 | 88 | (when nearly half was rubbed in) p. soft. |
| | 20 | 84 | affected with drowsiness, and complains of being cold; p. soft, eyes look heavy. |
| | 30 | 84 | complains of being cold and drowsy, p. soft. |
| | 40 | 84 | cold and drowsy; pulse weaker. |
| | 50 | 72 | feeble and small; countenance pale; coldness continues, and is accompanied with disagreeable sensations. |
| | 60 | 76 | weak, and not quite regular; complains of great weakness. |
| | 70 | 90 | regular and rather stronger. |
| | 90 | 92 | |
| | 120 | 96 | still complains of being cold; and the disagreeable sensations did not go off till after supper, when his appetite was better than usual. |
| | 180 | 108 | The friction was continued 40 minutes. |

The above experiment was made Dec. 11, at 6, p. m. and the following on the 13th, at 9, a. m. The preparation used was was the ſame as before, except that it contained one drachm more of the tincture of opium, namely, three drachms. The principal part was rubbed into the left leg and foot, and the remainder into the right. The application was made before he had breakfaſted, or uſed any exerciſe or exertion, and the liniment was made warm before it was applied.

| | Min. | Pulſe | |
|---|---|---|---|
| | — | 95 | immediately before the opium was applied. |
| In | 15 | 86 | ſoft, and eaſily compreſſed. |
| | 30 | 83 | weak and ſoft; complains of being very weak, cold and ſhivery; countenance looks ſickly; ſkin feels cold. |
| | 45 | 83 | ſoft; complains much of being cold all over, but particularly his feet; is ſhivery and very weak; ſpirits depreſſed; feels drowſy; eyes look dull. |
| | 60 | 90 | weak and ſmall; back painful; complains of being affected with a greater degree, and a different kind, of coldneſs, to any he ever experienced before. |

I now aſked if he had felt exhilarated at all, either in this or the former experiment? He anſwered, No; and could not help ſmiling at the queſtion, though he looked like one in the cold fit of an intermittent. He now requeſted to have his breakfaſt. I enquired whether he felt hungry? He replied, that he never was ſo hungry in his life. He ate his breakfaſt greedily, and was ſoon relieved from every diſagreeable ſenſation.

On his ſaying he had often been very cold, but had never before found that ſenſation accompanied with ſuch *diſagreeable* ſymptoms, I mentioned the word Horror, and he ſaid it conveyed the beſt idea of what he had felt.

[ To be continued. ]

